# Nurses’ perspectives on the role of relatives in emergency situations in nursing homes: a qualitative study from Germany

**DOI:** 10.1186/s12877-022-02991-y

**Published:** 2022-04-05

**Authors:** Sven Schwabe, Jutta Bleidorn, Andreas Günther, Nadia Primc, Giovanni Rubeis, Nils Schneider, Juliane Poeck

**Affiliations:** 1 Institute for General Practice and Palliative Care, Hannover Medical School, OE 5440, Carl-Neuberg-Str. 1, 30625 Hannover, Germany; 2 Institute of General Practice, University Hospital Jena, Jena, Germany; 3 Fire Department, City of Braunschweig, Braunschweig, Germany; 4 Institute of History and Ethics of Medicine, University of Heidelberg, Heidelberg, Germany; 5 Division Biomedical and Public Health Ethics, Karl Landsteiner University of Health Sciences, Krems an der Donau, Austria

**Keywords:** Nursing home, Qualitative methods, Relatives, Emergency, Geriatrics

## Abstract

**Background:**

In nursing homes, emergencies often result in unnecessary hospital transfers, which may negatively affect residents’ health. Emergency management in nursing homes is complicated by structural conditions, uncertainties and difficulties communicating with the treating healthcare professionals. The present study investigated the role played by relatives in this emergency management, as perceived by nursing staff.

**Methods:**

Within the context of a larger multi-method, interdisciplinary research project, we conducted six focus group discussions and 33 semi-structured interviews with nurses at nursing homes in northern Germany between September 2020 and April 2021. Discussions and interviews focused on emergency management in nursing homes, and were recorded, transcribed and analysed using qualitative content analysis, according to Mayring.

**Results:**

Nurses reported that relatives were actively involved in emergency management in the nursing homes. Relatives were informed when there was an emergency situation, and they participated in decision making around the resident’s care. Nurses sometimes perceived the involvement of relatives as challenging, due to a lack of time or staff, the opposing views of relatives and/or uncertain communication structures; however, they were willing to involve relatives according to the relatives’ preferences. The role played by relatives was seen to range from that of an active supporter to that of a troublemaker. On the one hand, relatives were reported to support nurses in emergency management (i.e. by identifying residents’ preferences and advocating for residents’ interests). On the other hand, relatives were often perceived by the nurses as overstrained and unprepared in emergency situations, leading them to override residents’ wishes, question the emergency plan and put pressure on the nurses’ decision making.

**Conclusions:**

Nurses perceive the roles played by relatives in emergency situations in nursing homes as relatively supportive or, alternatively, demanding and troublesome. The timely involvement of relatives in emergency planning, the establishment of clear agreements with general practitioners and the development of trusting relationships between nursing staff and relatives may improve emergency management for nurses.

## Background

Emergency situations in nursing homes frequently result in the unnecessary utilisation of emergency medical services and emergency department visits [1–5]. Often, responses to these situations do not comply with residents’ wishes; furthermore, they may interrupt residents’ continuity of care and increase the likelihood that residents will suffer from harmful effects, such as distress, disorientation, nosocomial infection and increased morbidity [6–8]. The most common reasons for emergency transfers from nursing homes are trauma after falling, altered mental status and infection [1]. Moreover, emergency management is frequently complicated by structural conditions, uncertainty and difficulties communicating with the treating healthcare professionals [9–12]. Lack of advance directives, unavailability of general practitioners and insufficient training, education and experience among nursing staff increase the likelihood of hospital transfers in these situations [13–15].

Nursing staff play a crucial role in emergency management, by assessing the potentially life-threatening situation, organising the initial treatment and determining whether further medical assistance is needed, all while caring for other residents [16, 17]. Communication and decision making with relatives is an important component of this process, because family inclusion is central to the provision of individualised care [18].

Research has shown that, when a person moves to a nursing home, relatives tend to be less involved in decision making [19]. However, when relatives stay involved and share information on the resident’s biography, meaningful activities and daily life preferences, the resident’s experience in the nursing home is generally improved [20]. From this perspective, nurses and other professional caregivers perceive relatives as a valuable resource for improving person-centred care [21, 22].

However, in the context of emergency management, relatives are primarily experienced as a challenge. Many studies have examined the reasons for hospital admissions (including unnecessary admissions) from nursing homes and identified relatives as a significant factor [23–27]. These studies have only reflected the problem-oriented perspectives of healthcare workers. In contrast, relatives report different experiences of emergency management in nursing homes, indicating a perceived responsibility to enforce residents’ wishes, an inclination to follow staff’s advice and a tendency to support healthcare professionals in their decision making; furthermore, they also report feeling insufficiently involved in treatment and transfer decisions [28]. From their perspective, relatives play a crucial und supportive role in emergency management; however, it remains unclear whether nursing staff agree with this perception – and to what extent. Accordingly, the present study explored nurses’ perspectives on the role played by relatives in emergency management in nursing homes.

## Methods

### Design

The multi-method, interdisciplinary research project NOVELLE aims at developing a concept for emergency management in nursing homes, based on an inter-professional process. In the present study, the results of two working packages of the larger research project (i.e. focus group discussions and semi-structured interviews) were combined and analysed separately, with respect to the role played by relatives in emergency situations. The aim of the in-depth interviews was to get a deep insight into the structures and processes of each nursing home and to explore ethical aspects of emergency management in nursing homes. The aim of focus groups was to develop a concept for emergency management considering the practical challenges of nursing staff in emergency situations. Ethical approval was granted by the Ethics Committee of Hannover Medical School (Nr. 8866_BO_K_2020; 27.01.2020) and Ostfalia University of Applied Sciences (02.07.2020). The study adheres to COREQ checklist for reporting qualitative research [29].

### Sampling and recruitment

Participants were recruited between September 2020 and January 2021, following a purposeful sampling strategy, in the northern German city of Braunschweig. In this process, representatives of all 23 nursing homes participating in the NOVELLE research project were approached via telephone or email, or in person. They were asked to nominate themselves or a proxy who was a certified nurse, nursing home manager or nursing home director with experience in emergency management in nursing homes. Nominees were invited to discuss their perceptions and management of emergency situations in nursing homes within either a focus group discussion or a semi-structured interview (depending on their preference). The semi-structured interviews were conducted in September and October 2020, and the focus group discussions took place between January and April 2021. The research team regularly informed participants about the study and data management. Written informed consent was provided by all participants.

### Data collection

Data were collected in two phases, according to: (1) the 33 semi-structured interviews (see Tables 1 and 2) the six focus group discussions (see Table 3). The in-depth interviews provided deep insight into the structures and processes of each nursing home and ethical aspects of emergency management, while the focus groups – which involved nurses from different nursing homes, as well as physicians and experts on medical ethics – illuminated differences in emergency management procedures. Participants could choose whether they prefer to join focus groups or participate in in-depth interviews.

**Table 1:** Participants of in-depth interviews

Number of Interviews	Function of participants	N
33	nursing care	14
	Residential area manager	14
	Nursing service manager	3
	Other	2
		33

**Table 2:** Topics of interview guide for semi-structured interviews

No.	Topics of interview guide
1	Personal professional experience and areas of responsibility
2	Definitions and interpretation of emergency situations
3	Decision-making and use of guidelines in emergency situations
4	Cooperation with other actors/professional groups in emergency situations
5	Challenges in dealing with emergency situations
6	Need for support in emergency situations
7	Consideration of residents‘wishes and personal will in emergency situations
8	General framework for good nursing care in emergency situations
9	Possibilities for implementing recommendations for action in emergency situations
10	Complementary factors in dealing with emergency situations

**Table 3:** Participants of focus groups

No.	Topic of the focus group	Function of participants	N
1	Emergency Situation „fall“	nursing care	1
		Nursing service manager	1
		Nursing home manager	1
2	Emergency Situation „derailed vital signs”	nursing care	3
		Nursing service manager	1
3	Further emergency management	Nursing home manager	1
		physicians	3
4	Assessment of emergency situation	Nursing service manager	1
		Experts in medical ethics	2
5	Emergency Situation „pain“	nursing care	3
		Nursing service manager	1
		Nursing home manager	1
6	Emergency Situation „breathlessness“	nursing care	4
		Nursing service manager	1
			24 (19 nursing staff)

With regards to the semi-structured in-depth interviews, 33 interviews took place in participants’ respective nursing homes. All interviews were conducted by two researchers (NP, GR) and lasted between 45 and 75 min. The mean age of participants was 39 years (range: 20–60). The interview guide addressed nurses’ perceptions and management of emergency situations, as well as decision making and ethical implications in this context. The interview guide consisted of 10 general topics (see Table 2) that in themselves contained over 40 different questions. Interviews continued until the sample size was sufficiently large and varied to address the study aims [30].

A total of 6 focus groups with 24 participants took place. As some nursing staff participated in several focus groups, the total number of participants in focus groups was 16 (including 11 nursing staff). The number of female participants was 13 (inducing 10 nursing staff). Due to COVID-19 restrictions, the focus group discussions were conducted via the video conferencing software BigBlueBotton, with a duration in the range of 92–122 min. The discussion guides addressed nurses’ perceptions and management of emergency situations in nursing homes. A general discussion guide was developed and adapted for each focus group (see Table 4). While physicians and experts on medical ethics were involved in two focus groups, only the nurses’ contributions were considered in the data analysis. All focus groups were moderated by the first and last authors (SS, JP).

**Table 4:** General discussion guide for focus groups

No.	Discussion guide
1	Please tell us about your experiences of emergency situations in your nursing home where … [a resident fell, resident’s vital signs were derailed, …]?
2.	Now, we would like to focus on single phases and challenges of emergency management.
2.1	How do the nurses’ experience, safety and qualifications affect emergency management?
2.2	How does staffing affect emergency management?
2.3	How does the accessibility of GPs and medical on-call service affect emergency management?
2.4	What role do relatives play in emergency management?
2.5	What role does resident will play in emergency management? What happens if the residents’ will is not known?
2.6	What other challenges do you face in emergency management?
3.	Now we would like to discuss with you, how to handle the challenges at emergency management.
3.1	How do you deal with insecure nursing staff?
3.2	How do you deal with insufficient staffing?
3.3	How do you deal with poor accessibility of GPs and medical on-call service?
3.4	How do you deal with challenging relatives?
3.5	How do you deal with it when you don’t know the residents’ will?
3.6.	How do you deal with [other challenges, see question 2.6]?

For both formats (i.e. focus group and interview), guides were developed, tested and discussed by the research team. All focus groups and interviews were digitally recorded and transcribed verbatim. Field notes were taken and provided valuable initial information for the development of the code tree in the data analysis.

### Data analysis

Data were analysed via a process of inductive and deductive coding, according to Mayring’s qualitative content analysis, using MAXQDA20 (qualitative data analysis software) [31]. In the first step, data from the focus group discussions and in-depth interviews were coded separately and independently by SS and JP (focus groups) and NP and GR (interviews). Within the research team, codings and codes were reviewed, discrepancies were discussed, and codings and codes were revised until consent has been achieved. In both coding procedures, the ‘role of relatives’ independently emerged as a sub-code. In the second step, sub-code content was merged and re-coded by SS and JP. The thematic coding and structuring of the data focused on relatives’ roles and functions in emergency management, from the perspective of nursing staff. A code tree was developed from the empirical data, and SS and JP independently allocated codes to thematic codes. In the final refined analysis, sub-themes were combined under thematic headings (see Table 5).

**Table 5:** Code tree

**Relatives as participants**	• Heterogeneity of relatives • Planning of emergency situations • Wish to be informed • Participation in decision making
**Relatives as supporters**	• Trustful communication • Trust in nurses’ recommendations • Mediation in conflicts with resident • Advocacy for resident • Support for general practitioners
**Relatives as troublemakers**	• Conflict with nurse’s recommendations • Conflict between resident’s will and relative’s wish • Conflict between relatives • Demanding relatives • Overstrained relatives

## Results

**Fig. 1:**
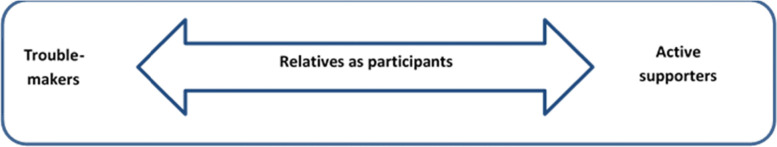
Roles of relatives in emergency situations

The analyses of the interviews revealed three key themes: (1) relatives as participants, (2) relatives as supporters and (3) relatives as troublemakers (see Fig. 1). The first theme was conceived of as the grouping that was most relevant to the study topic, whereas the second and third themes were conceptualised as theoretical extreme sub-types [32]. Empirically, these extreme types were rarely encountered in their pure form, but on a spectrum (see Fig. 1). The results are reported in the context of each thematic heading, supported by representative quotations.

### Relatives as participants in emergency situations

Nurses perceived no uniform role played by relatives, but a wide variety.

*Regarding the relatives, they are sometimes really different. (Nurse, I10: 150)*

Nevertheless, in both the interview and the focus group data, relatives of nursing home residents were considered important players in the planning, communication and decision making processes within emergency situations. When a nursing home takes on a new resident, nurses often involve relatives in planning for potential emergencies:

*Some residents don’t have advance directives. We are lucky to have a physician here who does advance care planning together with relatives and our nurses. Then, relatives have to decide: What would be the best option for my relative? This is a great support for us, because there we write down what to do to, if … (Nurse, I33:15)*

Irrespective of whether emergency planning has occurred, in emergency situations, nurses usually contact relatives. Relatives generally have an agreement in place with the nursing home as to the times and situations in which they wish to be informed (e.g. some do not want to be called at night).

*Usually relatives and general practitioners cooperate with us and want to be informed about emergencies. Then we describe the situation and they participate in decision making. (Nurse, FG5:266)*

In urgent and severe emergencies, the involvement of relatives is postponed in order to prioritise contact with emergency services. In these situations, nursing staff make decisions on behalf of the resident and inform relatives only afterwards.

*Then I actually call the emergency service first and do inform the relatives afterwards. I don’t need a relative’s decision here. Emergency is emergency. (Nurse, FG5:372)*

In the event of a resident’s limited capacity to consent (due to, e.g., neurodegenerative disease), nurses must involve relatives (or the resident’s proxy) in all decision making.

*Regarding the population of our residents here, most of them are not able to decide on their own. Then it’s up to the relatives, the patient’s proxy and us to decide what to do. (Nurse, FG6:85)*

### Relatives as active supporters

Nurses perceive relatives as active supporters when they agree with the nurses’ recommendations and are engaged in emergency management.

*We talk with the resident and with the relatives concurrently and we come to an agreement. In most of the cases, the relatives say: ‘Ok, if you assess the situation like you do, then we agree to have the situation monitored in the nursing home’. (Nurse, FG1:70)*

In these cases, relatives trust in the nurses’ competence and experience and follow their recommendations. Therefore, it is beneficial for nurses to prepare relatives for potential emergency situations and build trusting relationships with them.

If a resident does not agree with the necessary emergency treatment (e.g. transfer to hospital) and the nurses feel incapable of following the resident’s wish, they may involve relatives in the decision. Relatives sometimes have significant influence over residents and are able to convince them to follow the nurses’ recommendations.

*If a resident refuses to go to hospital and I think this is necessary, then I try to speak with the relatives. Mostly they follow my advice and discuss the topic with the resident once more. They sometimes have a higher impact than me. (Nurse, I26:39-43)*

If nurses deem hospital transfer necessary but the resident refuses, they may ask family members to accompany the emergency transport. This strategy can help to decrease the barrier for residents to go to the hospital and facilitate emergency management.

*If someone refuses emergency transport by all means, we sometimes call relatives to support us. This is of course not ideal, because then emergency service has to wait, and now, in times of corona [virus] this is currently not possible. But in the past, we called them, they came here and accompanied their relative to the hospital. This was much, much easier. (Nurse, I04: 155)*

On the other hand, relatives can also protect residents from receiving treatment that opposes their wishes. Even if emergency services have been called and an emergency physician is on site, nurses may defer to relatives’ decisions on whether to proceed with a hospital transfer.

*When we have a 96-year-old resident who is in a very bad condition, and death is forthcoming, but there was no [ … ] advance care planning, and emergency service wants to bring him to the hospital, then we call the relatives and say: ‘Here, please say that he refuses to go to hospital’. And usually emergency service follows the relatives’ wish. (Nurse, I04: 153)*

At times, nurses may try to involve a general practitioner in the emergency situation, either by asking them to come to the nursing home or by requesting medical advice. In these situations, relatives may call the general practitioner to emphasise the urgency of the situation.

*Sometimes it makes sense if relatives once again call the general practitioner. They emphasise the urgency and pressurise the general practitioner to a prompt answer. (Nurse, FG6: 141)*

### Relatives as troublemakers

Nursing staff perceive relatives as a challenge when they do not follow the nurses’ advice or the residents’ wishes, and when they are overstrained by the situation. In these situations, disagreements are likely to arise between nurses and relatives and, in some instances, relatives may pressure nurses to call an ambulance.

*Mostly, we see clearly that there is no emergency service necessary, but relatives insist on a hospital transfer. (Nurse, FG1:30)*

In other situations, relatives want the resident to stay in the nursing home, even if nursing staff do not consider this is a viable option.

*We had the case that a resident fell on her head, and she really had a concussion. For us it was clear that she needed a hospital transfer but the relatives asked us to keep her here. And even the physician shook his head [about the relatives’ request]. (Nurse, FG3: 45)*

In the event that differing opinions about emergency management exist between nurses and relatives, nurses are sometimes influenced by a threat of legal consequences.

*There is also the fear of our nursing home regarding legal consequences. If you do not react immediately then you are in trouble with the relatives. (Nurse, I16: 85)*

A complicated situation arises when a resident’s will is known and clearly written in an advance directive, but relatives cannot accept it. On the one hand, nurses feel obliged to respect the resident’s wishes; but on the other hand, they fear the consequences of doing so.

*I have a resident who has an advance directive, and everything is clear. But yet, I have relatives who cannot let him go: ‘Father mustn’t die!’ This is complicated, because they do not accept the resident’s will. I get in trouble, because if I follow the patient’s decree, then afterwards the relatives will accuse me of being responsible for his death. This is difficult. (Nurse, I10: 147-149)*

Although nurses emphasise the importance of emergency planning, they also acknowledge that emergencies are highly challenging for relatives. Sometimes, relatives’ experiences of feeling overburdened leads them to suddenly change their treatment plans.

*We frequently have relatives who cannot let the resident go. Actually, everything is clear and written down [about how to proceed in emergency situations], but when emergency situations occur they steer us in the opposite direction and say: ‘Please, once more!’ This is really challenging for us. (Nurse, I27: 114:120)*

## Discussion

In our analysis, three themes emerged relating to nurses’ perspectives on the role of relatives in emergency management in nursing homes: relatives as participants, relatives as supporters and relatives as troublemakers. The first theme pertained to the grouping that was most relevant to the study topic, whereas the second and third themes were conceptualised as theoretical extreme sub-types.

Previous studies on this topic have examined the causes of nursing home residents’ unnecessary hospital admissions. In most of these studies, demanding relatives have been reported to increase residents’ likelihood for hospitalisation and thus to represent a challenge for nurses and general practitioners [2–4, 8, 10, 33]. It is likely that this predominantly negative perspective on the role of relatives in emergency situations has been shaped by the studies’ problem-oriented research questions. In contrast, the object of the present study was to engage in a more open exploration of the role of relatives in emergency management in nursing homes, from the perspectives of nursing staff. The results confirmed that relatives play a crucial role in emergency management in nursing homes: nurses perceive them as active participants in the emergency situation and decision making; however, their participation may be experienced as relatively more supportive or, alternatively, demanding and troublesome.

Our results support previous findings on the important contribution made by relatives within the context of emergency management in nursing homes [18, 34]. For instance, Puurveen et al. identified a wide range of roles played by relatives in nursing homes, including the supply of hands-on assistance, the management of care and the provision of socio-emotional support to the nursing home community [34]. In emergency situations, the involvement of relatives is mainly limited to decision making, emergency management and advocacy [23]. Nurses try to inform relatives about emergencies as quickly as possible, in order to encourage their involvement from an early stage. A possible rationale for their doing so is that relatives often provide important information about residents’ wishes, medications and health [35, 36].

Several factors determine relatives’ level of involvement in nursing homes – particularly the related resident’s length of stay and physical condition, the relative’s relationship to that resident and the general conditions of the nursing home [37]. Furthermore, our results show that relatives’ degree of involvement also differs according to the emergency situation. Some relatives do not wish to be informed of an emergency during the night-time (opting instead to trust the nurses’ expertise); others want to participate in every step of the emergency management. The nurses in our study respected relatives’ differing levels of involvement and tried to act in accordance with their preferences.

In general, nursing staff in nursing homes have a positive but heterogeneous perception of relatives [22]. They perceive relatives as both time-consuming and demanding, as well as valuable resources for improving care [21, 38]. However, in the context of emergency management, nurses and other healthcare practitioners mainly experience relatives as a challenge. In these situations, relatives tend to have significant influence over the decision of whether or not to initiate a hospital transfer, and they may pressure nurses to initiate a transfer when it is actually unwarranted [1, 5, 39]. The literature reports that such pressure is predominantly exerted when relatives are lacking in confidence about the quality of care at the nursing home and unprepared for the resident’s end of life; it also arises when advance care planning and communication and agreement within the family regarding caring goals have not occurred [39]. Our findings confirm this pattern, insofar as relatives were reported to complicate emergency management when they were overburdened by the situation, unprepared for the resident’s imminent death and lacking trust in the nurses’ recommendations. In these situations, nurses viewed relatives as troublesome and were sometimes fearful of being accused by the relatives of causing the resident’s death; thus, they sometimes followed the relatives’ wish in order to avoid trouble and potential legal consequences.

In contrast to healthcare professionals’ problem-oriented perception of relatives, relatives tend to perceive themselves as advocates for residents with respect to the hospital transfer decision [24]. In our study, nurses agreed with this self-perception on the part of relatives, confirming that relatives often provide helpful information about residents’ presumed wishes, assist nurses in their efforts to involve general practitioners and emergency services and convince residents to follow the nurses’ advice. Furthermore, they are often able to speak to residents’ histories and desires, especially in cases in which residents are unable to communicate. Thus, it seems beneficial for nurses to invest in building trusting relationships with relatives at an early stage and incorporating relatives in treatment planning for possible emergencies.

However, even trusting relationships between relatives and nursing staff and advance communication about emergency situations are no guarantee for smooth management when such emergencies arise. Rather, relatives are often overburdened by emergency situations, with the result that they avoid conversations about the end of life situation and refuse to accept the resident’s forthcoming death [40, 41]. In our study, nurses reported that, even when emergency planning and communication about a resident’s forthcoming death are in place, relatives sometimes escalate the emergency management situation. This finding prevents us from overestimating the importance of planning and relationship-building and instead underlines the fact that emergency situations always develop according to their own dynamics.

There is growing evidence of the potential effectiveness of advance care planning in nursing homes [42–45]. Relatives have been identified as one of five key facilitators of this process. Our findings, reporting the perspectives of nurses, align with these reports in the literature. Indeed, relatives are likely to play a key role in both emergency and advance care planning. By identifying residents’ presumed wishes, they can improve patient autonomy and reduce stress for nurses during emergency situations.

### Strengths and limitations

The present study was based on a secondary analysis of two forms of qualitative data: focus group discussions and individual interviews. Different guidelines were applied and different interviewers were involved in the collection of both forms of data. Moreover, general practitioners and experts in medical ethics joined two of the six focus groups, and their presence may have influenced the nurses’ reports. To minimise this potential bias, we checked the focus group data against the individual interview data, finding similar topics and themes.

Focus groups and in-depth interviews took place during the COVID-19 pandemic and participants’ statements may have been influenced by COVID-specific experiences (i.e. restrictions of visiting and contact). However, COVID-19 has not been a topic in the interview guides and was not evaluated separately. We applied a purposive sampling strategy to recruit nursing homes and nurses for the study. All nursing homes in the region of intervention were approached, and participation was voluntary. It is possible that a positive selection bias occurred with respect to nursing homes practicing advanced emergency management.

A strength of the study is the broad data pool and the mixture of data from individual interviews and focus groups, which provided deep and broad insight into the nurses’ perceptions of relatives in emergency situations. As individual points and perceptions were discussed with other nurses in the focus groups, multi-perceptivity and reflexivity were ensured; thus, the validity of the results is high.

## Conclusions

Nursing staff perceive relatives’ roles in emergency management in nursing homes to be relatively supportive or, alternatively, disrupting and troublesome. Additionally, nurses respect and value relatives’ preferences with respect to the manner in which they are informed of emergency situations and the degree to which they participate in decision making in these situations. The timely involvement of relatives in emergency planning, the clarification of the role played by relatives in advocacy and decision making and the development of trusting relationships between nursing staff and relatives may improve emergency management for nurses.

## Data Availability

The datasets used and/or analysed during the current study are available from the corresponding author upon reasonable request.
